# Development of Membrane Selective Electrode for Determination of the Antipsychotic Sulpiride in Pharmaceuticals and Urine

**DOI:** 10.3390/s90604309

**Published:** 2009-06-03

**Authors:** Ma Soledad García, Joaquín A. Ortuño, Ma Isabel Albero, Mustafa Salem Abuherba

**Affiliations:** Department of Analytical Chemistry, Faculty of Chemistry, University of Murcia, 30071-Murcia, Spain; E-Mails: msgarcia@um.es (M.S.G.); jortuno@um.es (J.A.O.); mialbero@um.es (M.I.A.); abumusalem@yahoo.es (M.S.A)

**Keywords:** ion-selective electrode, sulpiride determination, pharmaceuticals, urine

## Abstract

The construction and electrochemical response characteristics of a poly(vinyl chloride) (PVC) membrane selective electrode for the determination of sulpiride (SPD) are described. The sensing membrane comprised an ion-exchanger formed between the protonated drug and tetraphenylborate (TPB^-^) in a plasticized PVC matrix. The influence of membrane composition on the electrode response was studied. The electrode showed a fast, stable and Nernstian response over a sulpiride concentration range (1 × 10^-4^ – 1 × 10^-2^ M) with a mean slope of 58.4 ± 0.9 mV dec^-1^ of concentration, a mean detection limit of 4.2 × 10^-5^ ± 1.2 × 10^-5^ M, a wide working pH range (2 – 8) and a fast response time (< 15 s). The electrode showed good selectivity towards sulpiride with respect to some inorganic and organic compounds. When the electrode was applied to the determination of sulpiride in pharmaceuticals and human urine, a high percentage of recovery was attained with no need for sample pretreatment procedures because of the lack of interfering matrix effects.

## 1. Introduction

Substituted benzamides are atypical neuroleptics and highly selective for dopamine receptors. The main pharmacological characteristics of these drugs include their ability to bind to a subgroup of dopamine D_2_ receptors, which may be located on the presynaptic terminal. Substituted benzamides were the first class of atypical anti-psychotics to be successfully used for schizophrenia and depression [1].

Sulpiride (SPD) 5-(aminosulfonyl)-*N*-[(1-ethylpyrrolidin-2-yl)methyl]-2-methoxybenzamide, a substituted benzamide with antipsychotic properties, acts as antagonist of the dopamine D_2_ receptors, a property which distinguishes from other antipsychotic agents. This particular feature may explain the very low incidence of side effects on the extrapyramidal system. It is used for treatment of psychopathological disorders, including neurosis, depression, schizophrenia, the psychopathology of senescence, anorexia, gastric or duodenal ulcers and irritable colon due to psychosomatic stress and various vertigo syndromes [2].

Typical pharmaceuticals which contain sulpiride include Dogmatil, Dolmatil, Sulpor and Guastil, in different forms, e.g. tablets, capsules, injectable ampoules, and suspensions containing 50 – 200 mg of sulpiride per unit.

Sulpiride is efficiently absorbed after oral administration and is eliminated principally by hepatic metabolism and subsequent urinary excretion. The normal dose of adults is 150 – 300 mg day^-1^ and for children 25 – 200 mg day^-1^. Its oral bioavailability is only 25 to 35%, with marked inter-individual differences. The peak plasma concentration is reached 4.5 hours after oral dosing. The usual half-life is 6 to 8 hours. Sulpiride is usually given in 2 or 3 divided doses and undergoes only limited metabolism: nearly 70 – 90 % of an intravenous injection and 15 – 20 % of an orally administered dose is excreted unchanged in urine [3]. Typical biological fluids examined for sulpiride concentrations are human serum and urine. Concentrations of sulpiride in the fluids of treated patients are in the ranges 0.03 – 0.6 and 10 – 360 μg mL^-1^, respectively

A review of the literature revealed that several analytical methods have been described for the determination of sulpiride in pharmaceuticals or biological fluids, including spectrophotometric [4-7], fluorimetric [8], chromatographic [9-14], electrophoretic [15-17], voltammetric [18] and chemiluminometric [19]; however, the methods proposed for the analysis of biological fluids suffer the inconvenience of time-consuming procedures and expensive instrumentation.

In recent decades potentiometric membrane ion-selective electrodes (ISEs) have been used in pharmaceutical and biological analyses [20-29] because these sensors offer the advantage of simple design and operation, low cost, fast response, low detection limit, adequate selectivity, good accuracy, wide concentration range, applicability to coloured and turbid solutions and possible interfacing with automated and computerized systems. However, a thorough literature survey has revealed no methods that use selective electrodes for the determination of sulpiride.

Tetraphenylborate derivatives have been used extensively in the composition of ion-selective electrode membranes. Although they can not form specific strong ion pairs they seem to play an active role as complexing agents [30,31]. Thus, the selectivity of some organic cations-selective electrodes based on tetraphenylborate derivates as charge carriers is significantly influenced by the charged carrier used.

The aim of this work was to develop a polymeric ion-selective electrode for sulpiride determination in pharmaceuticals, and human urine. The overall aim is to develop sensors for point-of-care clinical analysis in the treatment of mentally ill patients.

## 2. Experimental Section

### 2.1. Reagents and solutions

Poly(vinyl chloride) (PVC); 2-nitrophenyl octyl ether (NPOE); bis(2-ethylhexyl) sebacate (DOS); dibutylphtalate (DBP); tetrahydrofuran (THF); (±) sulpiride powder and sodium tetraphenylborate (NaTPB). Nanopure water (Resistivity in MΩ·cm at 25 °C = 18.2) prepared with a Milli-Q (Millipore) system was used throughout.

#### Standard sulpiride hydrochloride solution 5 × 10^-2^ M

Prepared by dissolving 1.707 g of pure drug in 0.5 mL of conc. HCl and diluting with water to 100 mL. Working solutions (1 × 10^-6^ to 2 × 10^-2^ M) were prepared by appropriate serial dilutions with acetic/acetate buffer solution of pH 4.7 and 2 × 10^-1^ M concentration.

#### Sodium tetraphenylborate 1 × 10^-2^M

Prepared by dissolving 0.3422 g of sodium tetraphenylborate to 100 mL with water.

#### Dosage form of sulpiride

Dogmatil 50 capsules (Sanofil-Synthelbe SA, Spain), contained 50 mg sulpiride, lactose, methylcellulose, talc, magnesium stearate and other excipients to total capsule weight; Dogmatil solution (Sanofil-Synthelbe SA, Spain): 500 mg sulpiride, sodium cyclamate, hydroxyethylcellulose, methylparaben, propylparaben, citric, hydrochloric and sorbic acids, lemon essence and water to 100 mL. Guastil pedriatic suspension (Uriach, Spain): 500 mg sulpiride, sacharose, sodium saccharin, microcrystalline cellulose, sodium carmelose, sodium chloride, methyl p-hydroxybenzoate, propyl p-hydroxybenzoate, strawberry essence and water to 100 mL.

### 2.2. Ion-exchanger preparation

The sulpiride tetraphenylborate (SPD-TPB) ion exchanger was prepared by reacting 25 mL of 2 × 10^-2^ M sulpiride hydrochloride solution with 50 mL 1 × 10^-2^ M sodium tetraphenylborate solution. The mixture was filtered through a porous number 4 sintered glass crucibles. The residue was first washed with distilled water until no chloride ion was detected in the washing solution and then with hexane before being dried at room temperature.

### 2.3. Construction and conditioning of the electrode

The membranes were prepared by dissolving 3.0 or 9.0 mg of SPD-TPB, 100 mg PVC and 200 mg of the plasticizer (NPOE, DOS or DBP) in 3 mL of tetrahydrofuran. This solution was poured into a Fluka glass ring (inner diameter 28 mm, height 30 mm) on a Fluka glass plate, and allowed to evaporate overnight. A 7 mm diameter piece was cut out with a Fluka punch for ion-selective membranes and incorporated into a Fluka electrode body ISE containing 1 × 10^-2^ M potassium chloride and 1 × 10^-3^ M sulpiride, and saturated with excess AgCl as internal filling solution. The composition of the different membranes assayed is shown in Table 1.

The electrodes were conditioned by soaking with constant stirring in a solution containing 1 × 10^-3^ M sulpiride in acetate/acetic buffer of pH 4.7 until the electrode provided a constant potential. When not in use, the electrode was kept immersed in the same solution.

### 2.4. Measurement system

**Figure 2.**
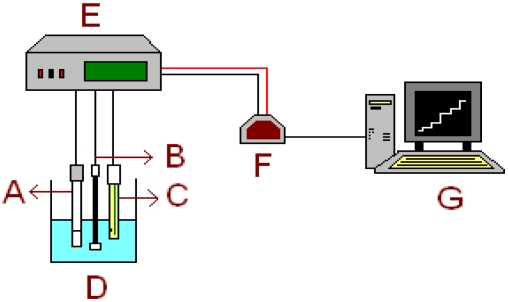
Measurement system used. A: Sulpiride selective electrode; B: Stirrer; C: Reference electrode; D: Sample; E: Potentiometer; F: Analogue-to-digital converter; G: Personal computer.

Potentials were measured with an Orion 960 Autochemistry System, the recorder output of which was connected to a personal computer, with acquisition program, via a DGH Corporation 1121 module analogue-to-digital converter (Manchester, UK). An Orion 90-02 double junction silver-silver chloride reference electrode containing 10 % (w/w) solution of KNO_3_ in the outer compartment and a Fluka electrode body ISE, were used. Figure 2 shows the measurement system used.

### 2.5. General procedure (calibration of the electrode)

Standard sulpiride solutions of 1 × 10^-6^ – 2 × 10^-2^ M in acetate/acetic buffer of pH 4.7 were prepared. The sulpiride-selective and reference electrodes were immersed and the potential of each sample solution was directly measured. The measured potentials were then plotted versus logarithmic values of concentrations and the calibration parameters were calculated by fitting calibration data to the equation shown in section *3.3*. For the dynamic response studies, the electrode was calibrated by injecting, while stirring, adequate small volumes of sulpiride standard solution in 50 mL of acetate/acetic buffer of pH 4.7 to obtain final concentrations in the range 1 × 10^-6^ – 1 × 10^-2^ M.

### 2.6. Procedure for the determination of sulpiride in dosage form

The content of sulpiride in capsules was determined by analysing five capsules separately. The powder content in each capsule was shaken with 0.5 mL conc. HCl and 5 mL of water. The mixture was then introduced into an ultrasonic bath for 5 min and diluted with water in a calibrated 10 mL flask. An accurately measured volume (100 μL - 2 mL) of this solution was diluted with acetic/acetate buffer of pH 4.7 in a calibrated 25 mL flask. For pharmaceuticals in solution or suspension, an accurately measured volume (100 μL – 2 mL) of this solution was directly taken and diluted to 25 mL with acetic/acetate buffer of pH 4.7. The potential of the different solutions was measured using the procedure described in section *2.5* and the SPD concentration was obtained by referring to a calibration plot obtained under identical experimental conditions for standard solutions of SPD. To validate the proposed method 500 μL aliquots of pharmaceutical solution samples, equivalent to 2.5 mg of SPD, to which different volumes (500 – 1,500 μL) of SPD 5 × 10^-2^ M solution were added, were diluted to 25 mL with acetic/acetate buffer of pH 4.7 and analyzed in triplicate by the potentiometric procedure described above.

### 2.7. Procedure for the determination of sulpiride in human urine

Urine samples containing different sulpiride concentrations were prepared by adding known amounts of sulpiride to 25 mL aliquots of blank urine samples of four volunteers, the sulpiride-selective and reference electrodes were immersed and the sulpiride concentration was determined by direct potentiometry using the standard additions technique.

## 3. Results and Discussion

### 3.1. Influence of membrane composition

**Figure 3.**
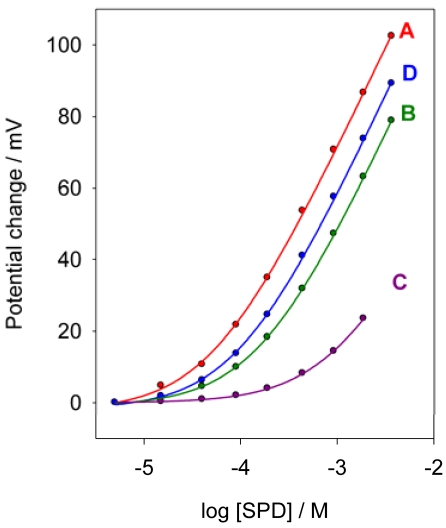
Calibration graphs of sulpiride obtained with the membranes A, B, C, D.

Four membranes of the different compositions (Table 1), prepared as described in the Experimental, were tested. Three plasticizers, with very different dielectric constants, were tested as membrane solvent, NPOE (ε = 23.9), DBP (ε = 6.4) and DOS (ε = 4). The calibration graphs obtained for the corresponding membranes, A, B and C, respectively, are shown in Figure 3.

As can be seen, the membrane plasticized with NPOE showed a better response and also presented a lower detection limit than the other two membranes, 3.7 × 10^-5^ M, 1.7 × 10^-4^ M and 4.1 × 10^-3^ M, respectively. As it is known, the detection limit of ISEs with dissolved ion exchangers is controlled by the analyte ion concentration present in the solution as a result of the distribution equilibrium of the ion pair between the membrane and the solution [32]. The solubility of the ion-pair in the organic solvent generally increases as the polarity and dielectric constant increases [33], which explain the lower detection limit obtained with NPOE as plasticizer. The NPOE was selected for further studies.

Two different ion exchanger SPD-TPB concentrations in the membrane were tested, 1.0 and 3.0 % (membranes A and D, respectively). The corresponding calibration graphs, Figure 3, show similar responses with both ion-pair concentrations, but the detection limit of membrane A (3.7 × 10^-5^ M) was lower than that of membrane D (1 × 10^-4^ M), due to the lower SPD concentration in the aqueous solution as a result of the distribution equilibrium of the ion-exchanger. Accordingly, the membrane A was selected for further studies.

### 3.2. Influence of pH

**Figure 4.**
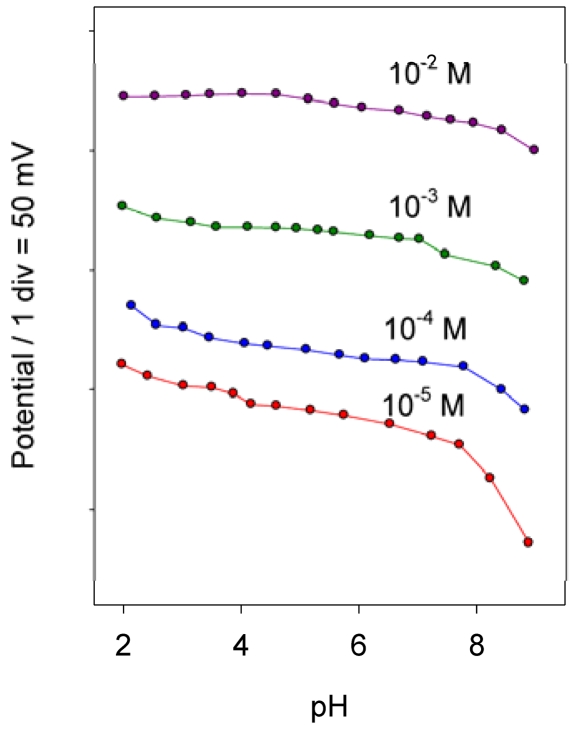
Influence of pH on the electrode potential for different sulpiride concentrations

The effect of pH on the electrode potential at various sulpiride concentration in the range 1 × 10^-5^ – 1 × 10^-2^ M was studied. The pH was varied by adding HCl or NaOH, and the results obtained are shown in Figure 4. As can be seen, the electrode potential was little influenced by pH in the range 2-8 for sulpiride concentrations between 10^-2^ – 10^-4^ M. At higher pH values, the potential decreased due to the gradual increase in the concentration of the deprotonated form of the SPD (pK_1_ = 8.9). In working with ion-selective electrodes for cationic drugs, we have observed some problems of precipitation of at high concentrations of the drug and higher pH values. A pH of 4.7 adjusted with 2 × 10^-1^ M acetic/acetate buffer was used for further studies.

### 3.3. Response characteristics

Ion-selective electrode characterization with a mathematical and computational program has been shown to be very useful for determining detection limits and selectivity constants [34]. Non-linear curve fitting using commonly available software was used for determination of the ISE characteristics [35]. The slope (S) and the detection limit (LOD) of the selected electrode were determined by fitting calibration data to equation:

$$E=E_{i}^{0}+S\log(\text{LOD}+c_{\text{SPD}})$$

The calibration parameters, evaluated from repeatedly making calibration graphs for sulpiride 1× 10^-6^ – 1 × 10^-2^ M, are shown in Table 2. As can be seen, a near-Nernstian response within a two decade concentration range, with low detection limit and good calibration reproducibility, was obtained.

### 3.4. Reproducibility

The repeatability of the calibration parameters was studied by making four successive calibrations with three different membranes 1, 2 and 3, cut out from the same original membrane, on the same day (n = 5).

The reproducibility on different days (n = 8) was studied with the three membranes and the reproducibility between the three membranes was obtained from the corresponding repeatability means. Table 3 shows the good results obtained in all the cases.

### 3.5. Response time

**Figure 5.**
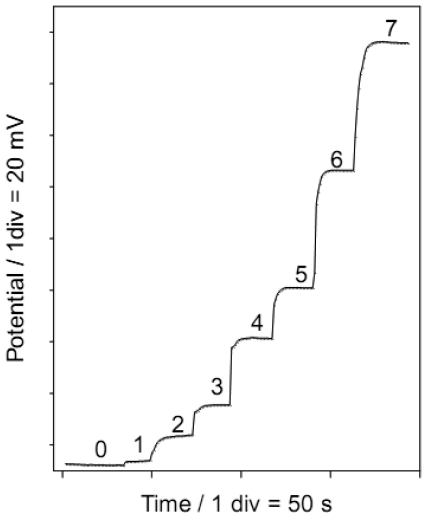
Dynamic response of electrode to different sulpiride concentrations. 0:0; 1:1 × 10^-6^ M; 2: 6 × 10^-6^ M; 3:1.6 × 10^-5^ M; 4: 6.6 × 10^-5^ M; 5: 1.6 × 10^-4^ M; 6: 1.1 × 10^-3^ M; 7: 9.1 × 10^-3^ M.

The dynamic response time is an important factor with selective electrodes. For the proposed ISE, the response time was obtained from the dynamic potential response corresponding to sulpiride concentration steps between 1 × 10^-6^ – 1 × 10^-2^ M (shown in Figure 5) by measuring the time required to reach 95 % equilibrium potential after increasing the concentration of the drug. The values obtained for different sulpiride concentrations are included in Table 2. The response time varied from 4 s for higher SPD concentrations and 15 s for lower concentrations.

The electrode lifetime was obtained by periodically performing calibration graphs for SPD and calculating the response slopes. The sulpiride selective electrode worked for at least 15 – 20 days, during which time no appreciable change in the calibration characteristics or response time was observed, while at higher times the slopes of the electrode started to decrease.

### 3.6. Selectivity

The selectivity of an ion-pair based membrane electrode depends on the physico-chemical characteristics of the ion-exchange process at the membrane-sample solution interface, on the mobility of the respective ions in the membrane and on the hydrophobic interactions between the ions and the organic membrane [36]. The selectivity of the SPD membrane electrode is related to the free energy of transfer of the SPD cation between aqueous and organic phases.

**Figure 6.**
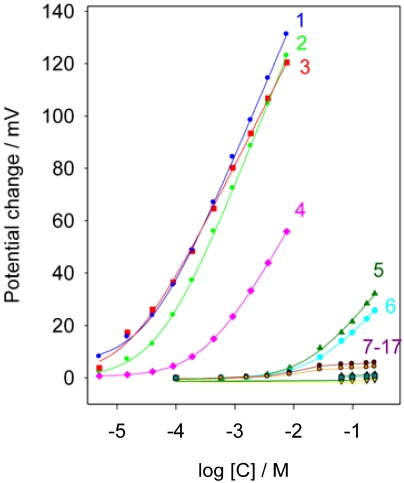
Calibration graph for sulpiride and interferent ions. Curves: **1** ranitidine; **2** sulpiride; **3** ofloxacine; 4 cimetidine; 5 K^+^; 6 NH_4_^+^; 7 – 17 Ca^2+^, Mg^2+^, glucose, lactose, sucrose, urea, uric acid, hipuric acid, amoxiciline, diclofenac and carbamazepine respectively.

The response of the electrode was studied toward several different substances: inorganic ions, organic species frequently present in pharmaceuticals and biological fluids, and other drugs used in pharmacological treatments (K^+^, NH_4_^+^, Ca^2+^, Mg^2+^, glucose, lactose, saccharose, urea, uric acid, hipuric acid, amoxicillin, cimetidine, ofloxacine, diclofenac, carbamazepine and ranitidine). Figure 6 shows the calibration graphs corresponding to the different species assayed. As can be seen, the electrode did not respond to Ca^2+^, Mg^2+^, glucose, lactose, sucrose, urea, uric acid, hipuric acid, amoxiciline, diclofenac or carbamazepine at concentrations lower than 2 × 10^-1^ M and to K^+^, NH_4_^+^ at concentrations lower than 1 × 10^-2^ M. The electrode gave a near Nernstian response to ranitidine, ofloxacine and cimetidine.

The selectivity coefficients of these three species were determined by applying the separate solution method comparing the concentrations that generate the same potential of the primary and interfering ion. The concentration of SPD that corresponds to the same potential observed for ranitidine, ofloxacine and cimetidine 1 × 10^-2^ M was calculated using its calibration graph and the selectivity coefficients were calculated with equation: K_SPD, J_ = C_SPD_/C_J_. The values obtained were 1.4; 0.9 and 0.06 respectively.

The selectivity coefficient for sodium was determined because this ion is present in high concentrations in urine. The value calculated from the potential measured in a 2 M NaCl solution was 10^-5.2^. Therefore, and taking into account the sodium and sulpiride concentration levels present in urine, no interference from sodium in the determination of sulpiride in urine is expected.

### 3.7. Analytical applications

The new sulpiride-selective electrode was satisfactorily applied to the determination of the drug in pharmaceuticals and human urine.

In the case of pharmaceuticals, the possible interference of different excipients and additives used frequently in pharmaceutical containing sulpiride was studied by adding different amounts of the possible interferent to samples containing 10^-3^ M SPD and applying the proposed method. No interference was observed in the presence of cellulose, saccharose, sorbic acid, sodium saccharine, magnesium stearate or talc, even at amounts higher than these contained in pharmaceuticals.

Table 4 shows the results obtained applying the proposed method to the dosage forms analyzed. The results obtained were in good agreement with the certified values.

The validity of the proposed method was confirmed by applying the standard addition technique to the pharmaceuticals analyzed. The results obtained are shown in Table 5. In all cases quantitative recoveries of between 99.1-100.8 % were obtained.

For the determination of SPD in human urine, possible interference from the sample matrix was previously studied. Four urine samples from different volunteers (urine blanks) were collected and the calibration graph for each was obtained by adding appropriate volumes of SPD 5 × 10^-2^ M to obtain a concentration of SPD between 2 × 10^-5^ – 7 × 10^-3^ M and applying the procedure described in *2.7*. No significant differences between the slopes and the detection limits corresponding to these calibrations were found and so the sulpiride concentration in urine samples was determined as described in Experimental without any pre-treatment procedure of the samples. In the absence of urine samples containing SPD, known amounts of SPD were added to blank urine samples and the results obtained are summarized in Table 6.

Good recoveries in all urine samples were obtained. The results obtained for different urines samples assayed were also compared by applying the *t*-test at the 95% confidence level. The calculated *t* value (0.52) did not exceed the theoretical value (2.20), indicating that there are no significant differences between the content of sulpiride added and it obtained by the proposed method.

Taking into account the normal dose and the pharmacokinetic of sulpiride mentioned in the Introduction, the concentration of SPD in urine passed over a 24 h period is within the range of SPD determination of the potentiometric method proposed.

## 4. Conclusions

The new ion selective electrode developed, based on a plasticized poly (vinyl chloride) (PVC) membrane containing the ion-exchanger formed between protonated sulpiride and tetraphenylborate, provides a rapid, sensitive, precise and inexpensive method for the direct potentiometric determination of sulpiride in pharmaceuticals and in human urine samples, in the physiological concentration range obtained after the usual therapeutic dose of sulpiride has been administered.

## Figures and Tables

**Figure 1.**
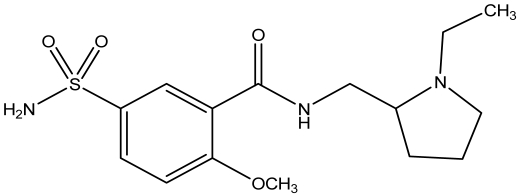
Sulpiride.

**Table 1.** Composition of the membranes.

**Percentage (w/w) of components in membranes**					
**Membrane**	**PVC**	**NPOE**	**DBP**	**DOS**	**SPD-TFB**
A	33.0	66.0	----	----	1.0
B	33.0	----	66.0	----	1.0
C	33.0	----	----	66.0	1.0
D	32.3	64.7	----	----	3.0

**Table 2.** Response characteristics of the sulpiride-selective electrode.

Slope (mV per dec) ± S.D.	57.5 ± 0.7
Linear range (M)	5 × 10^-5^ to 1 × 10^-2^
Detection limit (M) ± S.D.	3.7 × 10^-5^± 7.0 × 10^-6^
Response time (s) 10^-6^ – 10^-2^ (M)	t_95%_ ≤ 15
Working pH range (10^-6^ – 10^-2^ M)	2 – 8
Lifetime (day)	≥ 15

**Table 3.** Repeatability and reproducibility of sulpiride electrode.

**Membrane**	**Repeatibility**	

	S ± SD*	LOD ± SD*
1	59.4 ± 0.3	5 × 10^-5^ ± 2 × 10^-5^
2	58.3 ± 0.8	4.9 × 10^-5^ ± 0.3 × 10^-5^
3	57.6 ± 0.4	2.8 × 10^-5^ ± 0.5 × 10^-5^
	Reproducibility between days	
1	58 ± 2	4 × 10^-5^ ± 2 × 10^-5^
2	57 ± 3	4 × 10^-5^ ± 2 × 10^-5^
3	57 ± 1	1.8 × 10^-5^ ± 0.2 × 10^-5^
	Reproducibility between membranes	
1,2,3	58.4 ± 0.9	4 × 10^-5^ ± 1 × 10^-5^

* Mean ± SD (n = 5)

**Table 4.** Determination of sulpiride in pharmaceuticals.

**Sample**	**Sulpiride**		
	**Labeled**	**Found**^(*)^	**% Recovery**^(*)^
Dogmatil capsules	50 ^(a)^	50.10 ± 0.28 ^(a)^	100.2 ± 0.6
Dogmatil solution	50 ^(b)^	50.11 ± 0.24 ^(b)^	100.2 ± 0.5
Guastil suspension	50 ^(b)^	49.98 ± 0.58 ^(b)^	99.9 ± 1.2

(*) Mean ± SD (n = 5);

(a) SPD mg/capsule;

(b) SPD mg/10 mL.

**Table 5.** Determination of sulpiride in pharmaceuticals.

**Sample**	**Sulpiride**			

	**Labeled**	**Added**	**Found**	**% Recovery**^(*)^
Dogmatil capsules	50 ^(a)^	8.54^(a)^	8.58 ± 0.02	100.5 ± 0.2
		17.08^(a)^	17.18 ± 0.02	100.6 ± 0.1
		25.62^(a)^	25.49 ± 0.05	99.5 ± 0.2
Dogmatil solution	50 ^(a)^	8.54^(b)^	8.56 ± 0.06	100.3 ± 0.7
		17.08^(b)^	17.18 ± 0.04	100.6 ± 0.2
		25.62^(b)^	25.57 ± 0.01	99.8 ± 0.1
Guastil solution	50 ^(b)^	8.54^(b)^	8.61 ± 0.02	100.8 ± 0.3
		17.08^(b)^	16.92 ± 0.18	99.1 ± 1.1
		25.62^(b)^	25.59 ± 0.10	99.9 ± 0.4

(*) Mean ± SD (n = 5);

(a) SPD mg/capsule;

(b) SPD mg/10 mL.

**Table 6.** Determination of sulpiride in urine.

	**Sulpiride**		
**Urine**	**Content ^(a)^**	**Found ^(a)^**	**% Recovery**
1	23.86	23.41	98.1
	57.69	56.70	98.3
	125.3	124.7	99.5
2	23.86	23.25	97.5
	57.69	57.53	99.7
	125.3	126.2	100.7
3	23.86	24.17	101.3
	57.69	60.87	105.4
	125.3	124.7	105.4
4	23.86	24.82	104.0
	57.69	56.98	98.8
	125.3	126.3	100.8

a mg/L of urine.
